# Synthesis and molecular docking of novel non-competitive antagonists of GluK2 receptor

**DOI:** 10.1007/s00044-014-1171-1

**Published:** 2014-07-24

**Authors:** Agnieszka A. Kaczor, Tomasz Wróbel, Christiane Kronbach, Klaus Unverferth, Tomasz Stachal, Dariusz Matosiuk

**Affiliations:** 1 Department of Synthesis and Chemical Technology of Pharmaceutical Substances with Computer Modeling Lab, Faculty of Pharmacy with Division of Medical Analytics, Medical University of Lublin, ul. Chodźki 4A, 20093 Lublin, Poland; 2 School of Pharmacy, University of Eastern Finland, Yliopistonranta 1, 1627, 70211 Kuopio, Finland; 3 Biotie Therapie GmbH, Meissner Str. 191, 01445 Radebul, Germany

**Keywords:** Carbazole derivatives, Non-competitive GluK2 receptor antagonists, Indole derivatives, Kainate receptors

## Abstract

Here we present the synthesis, pharmacological activity, and molecular docking of novel non-competitive antagonists of GluK2 receptor. The compounds concerned are derivatives of indole and carbazole and are the second reported series of non-competitive antagonists of the GluK2 receptor (the first one was also published by our group). The activity of the indole derivatives is in the micromolar range, as in the case of the first series of non-competitive GluK2 receptor antagonists. We have found that designed carbazole derivatives are devoid of activity. Active indole derivatives interact with the transduction domain of the GluK2 receptor, i.e., the domain which links the transmembrane region of the receptor with the agonist-binding domain. The binding pocket is situated within one receptor subunit.

## Introduction

The glutamatergic system is an attractive molecular target for pharmacological intervention (Kaczor and Matosiuk, 2010). Ligands acting on ionotropic glutamate receptors (iGluRs: NMDA, AMPA, and kainate receptors) or metabotropic glutamate receptors (mGluRs) are potential drug candidates for the treatment of neurodegenerative diseases (Alzheimer’s disease, Parkinson’s disease, Huntington’s disease), epilepsy, as well as schizophrenia, anxiety, and memory disorders (Kew and Kemp, 2005). Although only a few glutamate receptor ligands have turned out to be clinically useful (firstly, because of the crucial role of the glutamatergic system in many physiological processes, and secondly, due to the unfavorable psychotropic side effects, traditionally linked with high-affinity NMDA receptor antagonists), ligands of kainate receptors seem to be especially promising. Kainate receptors are involved in epileptogenesis and inducing synaptic plasticity, mainly via the mossy fiber long-term potentiation mechanism. Thus, antagonists of kainate receptors are potential anti-seizure and neuroprotective agents. Moreover, non-competitive antagonists of AMPA receptors are well tolerated in preclinical and clinical studies (Szénási *et al*., 2008), thus it may be expected that this will also be the case for such ligands of kainate receptors.

**Fig. 1:**
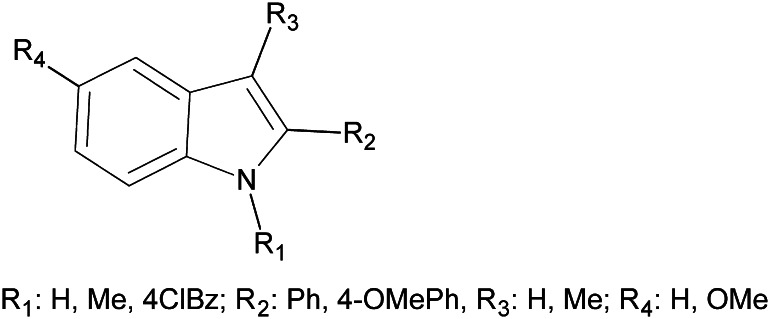
Non-competitive antagonists of GluK1/GluK2 receptors (Kaczor *et al*., 2012)

Research on non-competitive antagonists of kainate receptors is hindered by the fact that only three series of such compounds have been obtained up to now (Kaczor *et al*., 2012; Valgeirsson *et al*., 2003, 2004). Recently, we have reported 1,2,3,5-tetrasubstituted indole derivatives which are among the most active non-competitive antagonists of the GluK1 receptor and are the first known such ligands of the GluK2 receptor, Fig. 1 (Kaczor *et al*., 2012). We have also suggested a binding site for them in the receptor transduction domain (Kaczor *et al*., 2014) which was enabled by the construction of whole receptor models (Kaczor *et al*., 2008, 2009, 2014). Here we present further modifications, **2**–**7**, of the lead compound E099-25011, (1-ethyl-5-methoxy-2-(4-methoxyphenyl)-3-methylindole), **1**. The lead compound was identified by searching the internal databases of compounds at the Elbion Institute, Radebul, Germany. **1** is an analog of Zindoxifene, an anti-estrogen, tumor-inhibiting compound (Schneider *et al*., 1991). We have previously optimized compound **1** by changing substituents in positions 1, 2, 3, and 5 of the indole system (Fig. 1) (Kaczor *et al*., 2012, 2014). Compounds **3** and **5**–**7** were tested for their affinity to the GluK2 receptor, and compounds **3** and **5** were found to be non-competitive antagonists at this receptor. Furthermore, we show how novel non-competitive antagonists **3** and **5** of the GluK2 receptor interact with the transduction domain of the previously constructed homology model of this receptor (Kaczor *et al*., 2014).

## Materials and methods

### Chemistry

Reactions were routinely monitored by thin-layer chromatography (TLC) in silica gel (60 F_254_ Merck plates) and the products were visualized with ultraviolet light at 254 nm. All NMR spectra were acquired on a Bruker AVANCE III 600 MHz spectrometer equipped with a BBO Z-gradient probe. Spectra were recorded at 25 °C using chloroform as a solvent with a non-spinning sample in 5 mm NMR-tubes. High resolution mass spectra (HRMS) were recorded on a Bruker microTOF-Q II and processed using Compass Data Analysis software. The elementary analysis was performed using a Perkin-Elmer analyzer. Melting points were determined with Boetius apparatus and are uncorrected.

#### *5*-*methoxy*-*3*-*methyl*-*2*-*(2*-*thienyl)indole* (**2**)

Colorless crystalline needles (EtOH). This compound was prepared from 0.05 mol of 4-methoxyphenylhydrazine hydrochloride, 0.05 mol of 1-(2-thienyl)propan-1-one (2-propionylthiophene), 100 ml of anhydrous ethanol, and 10 ml of ethanol saturated with HCl, which were mildly boiled in a round-bottomed flask with a reflux condenser for 4 h. The reaction mixture was left overnight. The precipitation obtained was filtered and purified by crystallization from ethanol and repeated washing with n-hexane. Because of the tendency of the products to photooxidation, they had to be kept in the dark in a refrigerator. Yield: 69 %, mp 100–102 °C. ^1^H NMR (600 MHz, CDCl_3_) *δ* = 10.82 (s, 1H, NH), 7.47 (dd, *J* = 1.2, 5.3 Hz, 1H, H-para thienyl), 7.25 (d, *J* = 8.8, 1H, H-7), 7.19 (dd, *J* = 3.6, 5.3 Hz, 1H, H-meta thienyl), 7.11 (dd, *J* = 1.2, 3.6 Hz, 1H, H-ortho thienyl), 7.04 (d, *J* = 2.4, 1H, H-4), 6.93 (dd, *J* = 2.4, 8.8 Hz, 1H, H-6), 3.78 (s, 3H, 5-OMe), 2.29 (s, 3H, 3-Me); ^13^C NMR (125 MHz, CDCl_3_) *δ* = 151.93(C-5), 132.85 (C_ipso_ thienyl), 131.04 (C-7a), 127.33 (C-2), 124.76 (C-ortho thienyl), 124.05 (C-meta thienyl), 122.95 (C-para thienyl), 122.41 (C-3a), 113.91 (C-6), 110.74 (C-3), 110.23 (C-7), 100.68 (C-4), 55.95 (C-5-OMe), 9.65 (C-3-Me); HRMS (EI) *m*/*z*: 243.3278 C_14_H_13_NOS (calcd 243.3282); Anal. Calcd for C_14_H_13_NOS: C, 69.10; H, 5.38; N, 5.76; S, 13.18. Found: C, 69.16; H, 5.42; N, 5.74; S, 13.14.

#### 1-(2-thienyl)propan-1-one (2-propionylothiophene)

0.25 mol (32.53 g) of propionic acid anhydride and 0.2 mol (16.83 g) of thiophene were heated to 60 °C in a three-necked flask, equipped with a mechanic mixer, air condenser, and thermometer. Next, while still mixing, 1.10 g of 85 % orthophosphoric (V) acid was slowly added. Heating was continued for 2.5 h at 125 °C (with the mixture getting darker). After cooling, the mixture was washed with 50 ml of water and 100 ml of 10 % solution of sodium carbonate. The organic layer was dried with anhydrous sodium sulfate and subjected to vacuum distillation. The fraction boiling at 99–103/14 mmHg was collected. bp 88 °C/7 mmHg (Harthough and Kosak, 1947). Yield 12.90 g (46 %).

#### *5*-*methoxy*-*1,3*-*dimethyl*-*2*-*(2*-*thienyl)indole* (**3**)

Colorless crystalline needles (EtOH). This compound was prepared from 0.4 g of sodium hydride (50 % oil suspension) and 10 ml of anhydrous DMF, which were placed in a three-necked round-bottomed flask, equipped with a mechanic mixer and a thermometer. The mixture was cooled to 0 °C, and then a solution of 0.001 mol of 5-methoxy-3-methyl-2-(2-thienyl)indole (**2**) in 10 ml of anhydrous DMF was added dropwise. The mixture was stirred for 45 min, and a solution of 0.001 mol of methyl sulfate in 5 ml of anhydrous DMF was added. After 20 min, the ice bath was removed and the mixing was continued for 1.5 h at room temperature. Then a few milliliters of water were carefully added to decompose the excess of sodium hydride. The reaction mixture was filtered, the filtrate was cooled, and 20 ml of water was added to it. The precipitation obtained was purified by crystallization from ethanol and repeated washing with n-hexane. Yield 41 %, mp 71–73 °C. ^1^H NMR (600 MHz, CDCl_3_) *δ* = 7.43 (dd, *J* = 1.2, 5.3 Hz, 1H, H-para thienyl), 7.21 (d, *J* = 8.8 Hz, 1H, H-7), 7.15 (dd, *J* = 3.6, 5.3 Hz, 1H, H-meta thienyl), 7.08 (dd, *J* = 1.2, 3.6 Hz, 1H, H-ortho thienyl), 7.01 (d, *J* = 2.4 Hz, 1H, H-4), 6.89 (dd, *J* = 2.4; 8.8 Hz, 1H, H-6), 3.74 (s, 3H, 5-OMe), 2.25 (s, 3H, 3-Me), 1.24 (s, 3H, 1-Me); ^13^C NMR (125 MHz, CDCl_3_) *δ* = 152.09 (C-5), 132.83 (C_ipso_ thienyl), 131.36 (C-7a), 128.71 (C-2), 122.53(C-ortho thienyl), 123.12 (C-meta thienyl), 123.08 (C-para thienyl), 121.69 (C-3a), 113.18 (C-6), 110.77 (C-3), 110.25 (C-7), 100.73 (C-4), 56.03 (C-5-OMe), 15.42 (N1-Me), 9.61 (C-3-Me); HRMS (EI): *m*/*z* 257.3552 C_15_H_15_NOS (calcd 257.3553); Anal. Calcd for C_15_H_15_NOS: C, 70.01; H, 5.87; N, 5.44; S, 12.46. Found: C, 69.95; H, 5.92; N, 5.48; S, 12.41.

#### *1*-*(1H*-*Indol*-*3*-*yl)*-*3*-*phenylprop*-*2*-*en*-*1*-*one* (**4**)

Derivative **4** was obtained by means of Friedel–Crafts acylation according to (Guchhait *et al*., 2011) in 7.5 % yield as a yellowish white solid; mp 225-230 °C. Spectral data according to (Guchhait *et al*., 2011).

#### *3*-*[1*-*(4*-*chlorobenzyl)*-*1H*-*indol*-*5*-*yl]*-*1*-*phenylprop*-*2*-*en*-*1*-*one* (**5**)

Yellowish solid (EtOH). This compound was prepared as follows: 0.01 mol of derivative **4** and 30 ml of anhydrous DMF were mixed in a round-bottomed flask equipped with a thermometer and a dropping funnel. The reaction mixture was cooled to 0 °C and 0.8 g of sodium hydride was added (50 % oil suspension). After 30 min of mixing, a solution of 0.012 mol of 4-chlorobenzyl chloride in 20 ml of anhydrous DMF was added dropwise. The reaction was continued at room temperature for 3 h. The mixture was filtered and 10–15 ml of water was added to the filtrate. The resulting resin-like substance was removed and the next portion of water (25–30 ml) was added until the solution becomes opaque. The mixture was kept in refrigeration for two hours and the precipitation obtained was filtered and purified by crystallization from ethanol and repeated washing with n-hexane. Yield 72.0 %, mp 235–238 °C, ^1^H NMR (600 MHz, CDCl_3_) *δ* = 8.64–8.54 (m, 1H, H-5), 7.92 (s, 1H, H-2), 7.85 (d, *J* = 15.5 Hz, 1H, H-4), 7.65 (s, 2H, CH), 7.46–7.26 (m, 9H, Ar, phenyl + benzyl), 7.13–7.09 (m, 2H, H-6, H-7), 5.35 (s, 2H, CH_2_); ^13^C NMR (151 MHz, CDCl_3_) *δ* = 184.47 (CO), 141.40 (C-2) 140.71 (C_ipso_ phenyl), 137.11 (CHCHCO), 135.34 (C-7a), 134.51 (C_ipso_ benzyl), 134.37 (C-para benzyl), 134.17 (C-ortho phenyl), 129.94 (C-para phenyl), 129.29 (C-meta benzyl), 128.88 (C-meta phenyl), 128.21 (CHCHCO), 127.09 (C-ortho benzyl), 123.97 (C-3a), 123.85 (C-6), 123.24 (C-5), 122.98 (C-4), 118.43 (C-3), 110.13 (C-7), 50.20 (CH_2_). HRMS (EI): *m*/*z* 371.8434 C_24_H_18_NOCl (calcd 371.8591); Anal. Calcd for C_24_H_18_NOCl C, 77.51; H, 4.87; N, 3.77; Cl, 9.53. Found: C, 77.55; H, 4.88; N, 3.73; S, 9.49.

#### *9H*-*4*-*oxo*-*1,2,3,4*-*tetrahydrocarbazole* (**6**)

A solution of 0.1 mol of phenylhydrazine in 150 ml of water was added dropwise for 1.5 h to a solution of 1,3-cyclohexadione in 100 ml of water. The orange precipitation of 1,3-cyclohexadione monophenylhydrazone obtained was filtered. Yield 99 %, mp 173.5 °C (Hester, 1969). 100 g of polyphosphoric acid (PPA) was heated to 80 °C and then 0.025 mol of monophenylhydrazone of 1,3-cyclohexadione was added. The temperature slowly increased to 110 °C due to an exothermic reaction. The reactants were mixed for 30 min and then the reaction mixture was poured onto ice. The precipitation obtained was filtered and crystallized from methanol. Derivative **6** was obtained in a 61.6 % yield as a colorless solid, mp 234–235 °C. Spectral data as described by (Rodriguez *et al*., 1989).

#### *9*-*(4*-*chlorobenzyl)*-*4*-*oxo*-*1,2,3,4*-*tetrahydrocarbazole* (**7**)

Colorless solid (EtOH). This compound was prepared as follows: 25 ml of DMF, 0.1 ml of water, and 0.013 mol of potassium hydroxide were mixed for 5 min. 0.01 mol of **6** was added and mixing was continued for 1 h. Then a solution of 0.0015 mol of 4-chlorobenzyl chloride in 10 ml of DMF was added dropwise and the reaction was continued under stirring for 2 h. The reaction mixture was kept in a refrigerator overnight. 5 ml of water was added and the first portion of precipitation was obtained and filtered. The second portion of precipitation was obtained after adding a further 15 ml of water. The combined precipitation was crystallized from ethanol. Yield 87.7 %, mp 171–173 °C. ^1^H NMR (500 MHz, CDCl_3_) 7.58 (d, 1H, *J* = 7.8, H-5), 7.33 (d, 1H, *J* = 8.0, H-8), 7.22 (dd, 1H, *J* = 7.2; 8.0, H-7), 7.20 (d, 2H, *J* = 8.4, H-meta benzyl), 7.13 (dd, 1H, *J* = 7.2;7.8, H-6), 6.87 (d, 2H, *J* = 8.4, H-ortho benzyl), 5.17 (s, 2H, CH_2_), 2.90 (m, 2H, H-1), 2.59 (m, 2H, H-3), 2.25 (m, 2H, H-2), ^13^C NMR (100 MHz, CDCl_3_) 163.32 (CO), 158.25 (C-9a), 148.73 (C_ipso_ benzyl), 139.25 (C-8a), 127.81 (C-para benzyl), 123.97 (C-meta benzyl), 123.85 (C-ortho benzyl), 116.08 (C-4b), 115.64 (C-7), 113.97 (C-6), 112.82 (C5), 111.09 (C-4a), 105.46 (C-8), 20.95 (CH_2_), 13.51 (C-3), 13.05 (C-1), 12.73 (C-2). HRMS (EI) *m*/*z*: 309.7822 C_19_H_16_NOCl (calcd 309.7890); Anal. Calcd for C_19_H_16_NOCl C, 73.66; H, 5.21; N, 4.52; Cl, 11.44. Found: C, 73.65; H, 5.22; N, 4.53; S, 11.41.

### Pharmacology

The pharmacological studies were performed as described previously (Kaczor *et al*., 2012). HEK293 lines expressing GluK2 kainate receptors, together with aequorin, a bioluminescent Ca^2+^ reporter protein, were used to determine the effect of the compounds being investigated on GluK2 receptor activity. The influx of Ca^2+^ ions through open kainate receptor ion channels led to oxidation of coelenterazine, the cofactor of aequorin, which eventually resulted in the emission of photons. After incubation of the cells with coelenterazine, the culture medium was replaced with an assay buffer (Ringer buffer + 100 mM CaCl_2_). In a luminometer (LumiStar, BMG, Germany), 275 μM of glutamate was applied to the cells and the luminescence signals were recorded before, during, and after glutamate application.

### Molecular modeling

The homology model of the GluK2 receptor was constructed as described previously (Kaczor *et al*., 2014). The crystal structure of the AMPA GluA2 receptor (PDB ID: 3KG2) (Sobolevsky *et al*., 2009) was selected as the main template. Additional templates were used for the *N*-terminal domain (crystal structure of the GluK2/GluK5 NTD tetramer assembly, PDB ID: 3QLV) (Kumar *et al*., 2011) and the ligand-binding domain (crystal structure of GluK1 ligand-binding domain (S1S2) in complex with an antagonist, PDB ID: 4DLD) (Venskutonytė *et al*., 2012). Homology modeling was carried out with Modeler v. 9.11 (Eswar *et al*., 2006). Input conformations of the compounds being investigated were prepared using the LigPrep protocol from the Schrödinger Suite. To sample different protonation states of the ligands in physiological pH, the Epik module was used. The structural and electronic parameters of the ligands were calculated with VegaZZ v.2.4.0.25 (Pedretti *et al*., 2004), Gausian09 (Frisch *et al*., 2009), and Discovery Studio 3.1. Molecular docking was performed with Glide from the Schrödinger Suite. Molecular dynamics of ligand-receptor complexes were performed as described previously (Kaczor *et al*., 2014). Ligand-receptor complexes were inserted into a POPC lipid bilayer and water with a suitable module of Schrödinger suite of programs, and sodium and potassium ions were added to balance the protein charges and then up to a concentration of 0.15 M. The stability of the ligand-receptor complexes was assessed by molecular dynamics simulations with Desmond v. 3.0.3.1 (Bowers *et al*., 2006) The ligand-receptor complexes in lipid bilayer were minimized and subjected to MD first in the NVT ensemble for 1 ns and then in the NPT ensemble for 20 ns. The following software was also used to visualize the results: Chimera v.1.5.3 (Pettersen *et al*., 2004), VegaZZ v.2.4.0.25, Yasara Structure v.11.9.18 (Krieger and Vriend, 2002), and PyMol v.0.99 (The PyMOL Molecular Graphics System, Version 0.99, Schrödinger, LLC).

## Results and discussion

### Chemistry

**Fig. 2:**
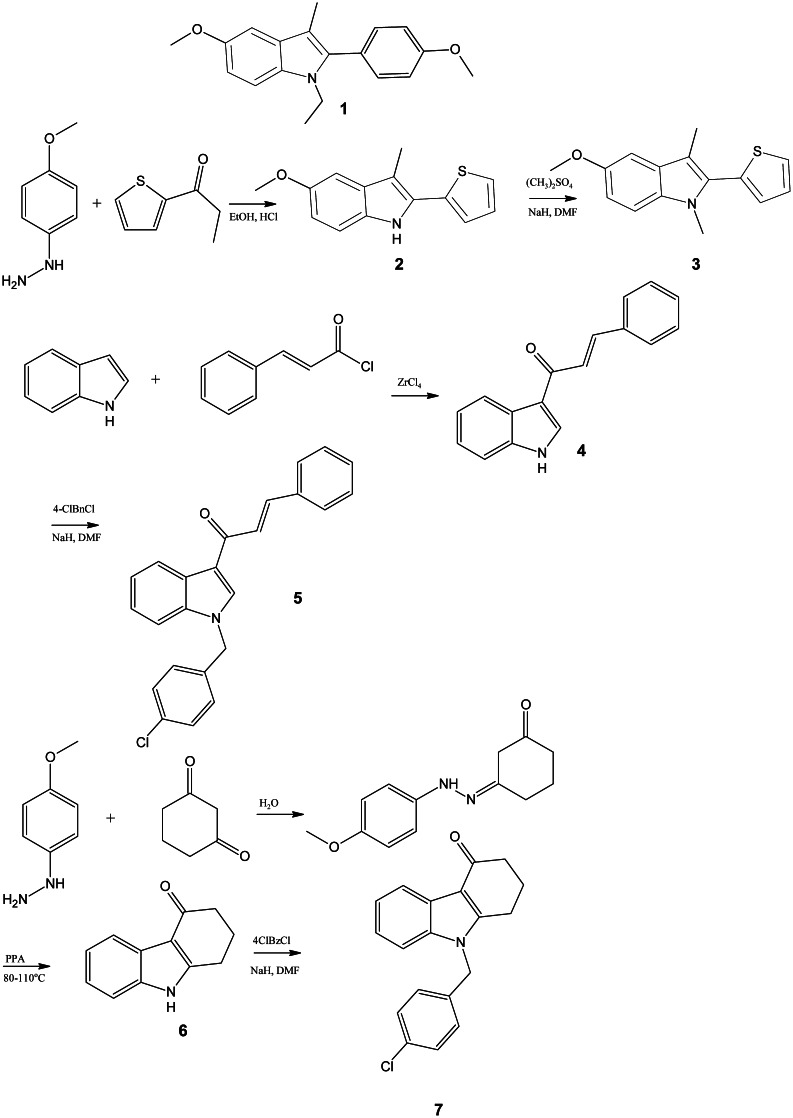
Scheme of reactions

The synthesis of compounds **2**–**7** is presented in Fig. 2. Compound **2** was obtained by Fischer indolization reaction. Alkylation of **2** with dimethyl sulfate resulted in compound **3**. The lead compound **1** and derivative **2** were previously characterized as anti-estrogens (Masatoshi *et al*., 1993; von Angerer *et al*., 1984, 1987, 1990). Compound **3** is a new compound. Compound **4** was obtained in Friedel–Crafts acylation of indole as previously described (Guchhait *et al*., 2011). Derivative **5** is a new compound and was obtained in alkylation of **4** with 4-chlorobenzyl chloride. Compound **6** was obtained by cyclization of monophenylhydrazone of 1,3-cyclohexadione (obtained from phenylhydrazine and 1,3-cyclohexadione) in PPA and was characterized previously (Rodriguez *et al*., 1989). Compound **7** is a new compound and was obtained by alkylation of **6** with 4-chlorobenzyl chloride.

### Pharmacology

Compounds **3** and **5**–**7** were tested for their affinity to GluK2 receptors as described previously (Kaczor *et al*., 2012; 2014). The IC_50_ values for the compounds being investigated are listed in Table 1. The investigations with the ^3^H-kainate binding assay showed no inhibition, which makes it possible to conclude that the antagonism for compounds **3** and **5** is of the non-competitive type.

**Table 1:** Pharmacological activity of novel ligands

Compound	GluK2 IC_50_, μM
**1**	0.7
**3**	12.0
**5**	1.7
**6**	100
**7**	22 % at 100 μm

## Structural and electronic parameters of novel ligands

In order to address the structure–activity relationship observed, structural and electronic parameters were calculated for compounds **1**, **3**, **5**, **6**, and **7**. The data are presented in Tables 2 and 3. The data shown in Table 2 show that the lack of activity of compound **6** may be explained by the fact that the molecular volume is too low and the dipole moment too high. The significant difference between the HOMO and LUMO values (Table 3) indicates that the compounds are nucleophilic and may participate as acceptors (through oxygen atoms) in hydrogen bonds with the binding pocket residues; this is in agreement with our earlier studies (Kaczor *et al*., 2012). Moreover, the novel ligands have more favorable lipophilicity values in comparison to the previous series, with the exception of compound **5** (Kaczor *et al*., 2012).

**Table 2:** Structural parameters of novel ligands

Compound	Surface, Å^2^	Ovality	Volume, Å^3^	Dipole moment, D
**1**	557.80	1.6637	324.86	3.97
**3**	485.2	1.5612	232.00	3.12
**5**	642.50	1.7163	335.30	3.89
**6**	379.00	1.4094	171.10	4.92
**7**	528.50	1.6128	274.00	3.95

**Table 3:** Electronic and physicochemical parameters of novel ligands

Compound	E_HOMO_, eV	E_LUMO_, eV	Lipophilicity
**1**	−8.03	0.04	4.94
**3**	−8.10	−0.33	4.65
**5**	−8.66	−0.52	6.44
**6**	−8.59	−0.14	2.51
**7**	−8.57	−0.39	4.96

## Ligand-receptor interactions

**Fig. 3:**
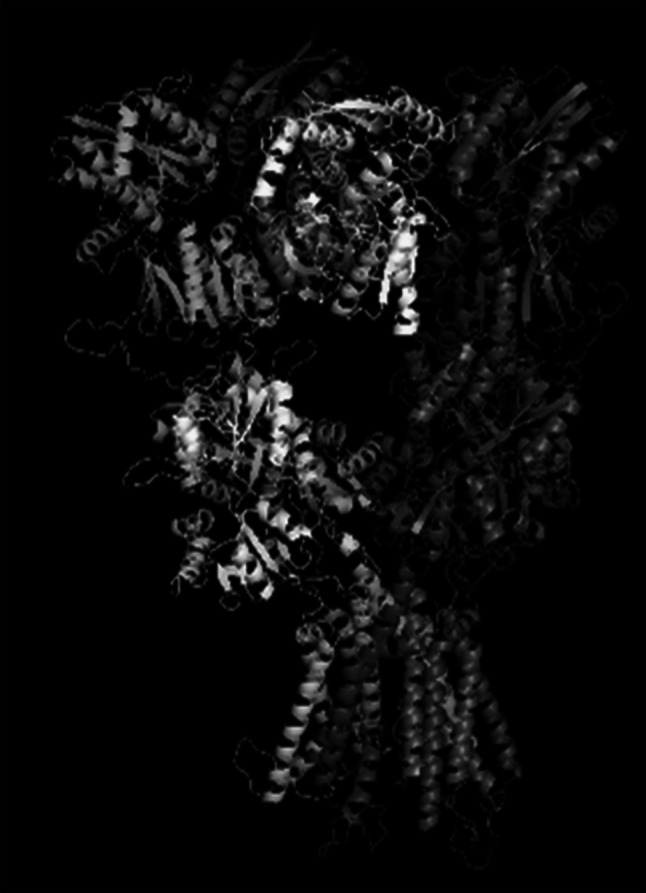
Model of the GluK2 receptor (Kaczor *et al*., 2014)

**Fig. 4:**
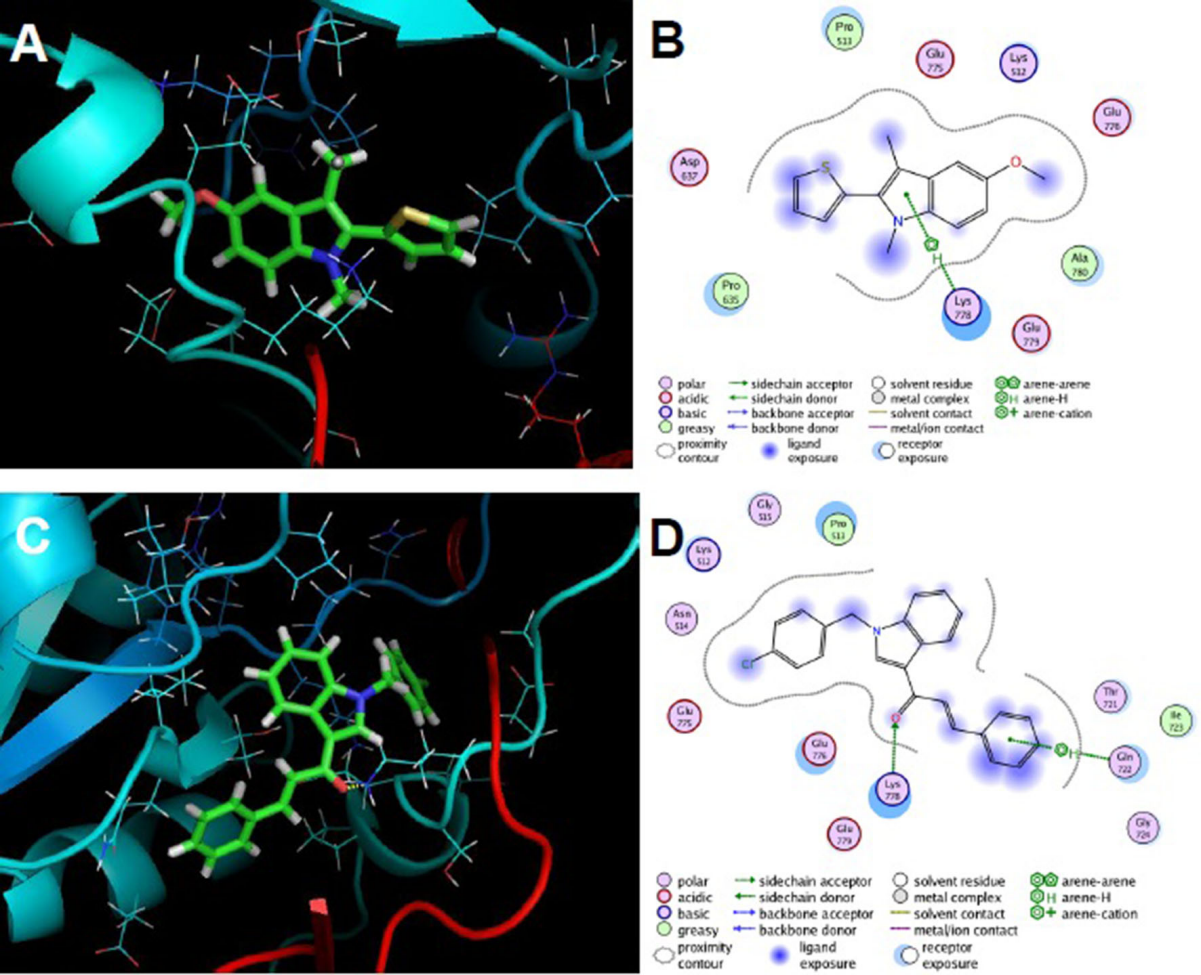
Compounds **3** (**a**, **b**) and **5** (**c**, **d**) in the binding pocket of the GluK2 receptor (transduction domain). **a**, **c**—overview of the binding pocket. **b**, **d**—schematic representation of the binding pocket

The binding site for non-competitive GluK2 receptor antagonists was identified in the receptor transduction domain, i.e., in the domain which connects the ligand-binding domain and the transmembrane domain (Fig. 3). This assumption was made on the basis of studies by (Balannik *et al*., 2005) for AMPA receptors as well as on our earlier molecular modeling studies (Kaczor *et al*., 2009, 2014). The exact binding site was found on the basis of sequence differences between the GluK1 and GluK2 receptors in the transduction domain as reported in our previous studies (Kaczor *et al*., 2009, 2014). There are no differences in the S1-M1 linker and in the S2-M4 linker. Asp823 and Asn824 in GluK1 correspond to Glu808 and Ser809 in GluK2. The interactions of compounds **3** and **5** with the GluK2 receptor are presented in Fig. 4a, b, c, d, respectively. There are the following residues in the binding pocket: Lys544, Pro545, Asn546, Gly547, Pro667, Asp669, Glu807, Glu808, Lys810, Glu811, and Ala812 which interact with both ligands. Furthermore, in the case of ligand **5**, the pocket is extended with the following additional residues: Thr753, Gln754, Ile755, and Gly756. The carbonyl group of ligand **5** forms a hydrogen bond with the side chain of Lys810. The binding pocket is situated within one receptor subunit which is in accordance with our recent studies (Kaczor *et al*., 2014).

## Conclusions

In this paper, we have reported the second series of GluK2 receptor non-competitive antagonists. We obtained two indole derivatives with activity in the low micromolar range. Furthermore, we found that the designed carbazole derivatives were not active. The novel non-competitive antagonists interact with the transduction domain of the GluK2 receptor, in the same way as the previously reported series. The binding site is located within one receptor subunit.
